# Template-based Quality Assessment of the Doppler Ultrasound Signal for Fetal Monitoring

**DOI:** 10.3389/fphys.2017.00511

**Published:** 2017-07-18

**Authors:** Camilo E. Valderrama, Faezeh Marzbanrad, Lisa Stroux, Gari D. Clifford

**Affiliations:** ^1^Department of Mathematics and Computer Science, Emory University Atlanta, GA, United States; ^2^Department of Electrical and Computer Systems Engineering, Monash University Melbourne, VIC, Australia; ^3^Department of Engineering Science, Institute of Biomedical Engineering, University of Oxford Oxford, United Kingdom; ^4^Department of Biomedical Informatics, Emory University Atlanta, GA, United States; ^5^Department of Biomedical Engineering, Georgia Institute of Technology Atlanta, GA, United States

**Keywords:** doppler ultrasound, empirical mode decomposition, fetal monitoring, dynamic time warping, sample entropy, signal quality

## Abstract

One dimensional Doppler Ultrasound (DUS) is a low cost method for fetal auscultation. However, accuracy of any metrics derived from the DUS signals depends on their quality, which relies heavily on operator skills. In low resource settings, where skill levels are sparse, it is important for the device to provide real time signal quality feedback to allow the re-recording of data. Retrospectively, signal quality assessment can help remove low quality recordings when processing large amounts of data. To this end, we proposed a novel template-based method, to assess DUS signal quality. Data used in this study were collected from 17 pregnant women using a low-cost transducer connected to a smart phone. Recordings were split into 1990 segments of 3.75 s duration, and hand labeled for quality by three independent annotators. The proposed template-based method uses Empirical Mode Decomposition (EMD) to allow detection of the fetal heart beats and segmentation into short, time-aligned temporal windows. Templates were derived for each 15 s window of the recordings. The DUS signal quality index (SQI) was calculated by correlating the segments in each window with the corresponding running template using four different pre-processing steps: (i) no additional preprocessing, (ii) linear resampling of each beat, (iii) dynamic time warping (DTW) of each beat and (iv) weighted DTW of each beat. The template-based SQIs were combined with additional features based on sample entropy and power spectral density. To assess the performance of the method, the dataset was split into training and test subsets. The training set was used to obtain the best combination of features for predicting the DUS quality using cross validation, and the test set was used to estimate the classification accuracy using bootstrap resampling. A median out of sample classification accuracy on the test set of 85.8% was found using three features; template-based SQI, sample entropy and the relative power in the 160 to 660 Hz range. The results suggest that the new automated method can reliably assess the DUS quality, thereby helping users to consistently record DUS signals with acceptable quality for fetal monitoring.

## 1. Introduction

Although medical care has reduced mortality rates across the globe, birth has still remained an event of extreme risk. Approximately 2.6 million stillbirths and 2.8 million early neonatal deaths occur each year (World Health Organization, 2016a,b). Different factors contribute to this high burden, such as the lack of specialized medical professionals and the high cost of the medical devices, mainly affecting low and middle income countries (LMICs). Leading causes for perinatal mortality include Intrauterine Growth Restriction (IUGR) and congenital abnormalities of which, Congenital Heart Disease (CHD) is the most common (van der Linde et al., 2011; Gardosi et al., 2013; Lawn et al., 2016). These abnormalities are currently being detected using ultrasound imaging and more specifically, fetal echocardiography is performed for CHD diagnosis. However, these techniques are expensive and can only be performed by trained sonographers or physicians; hence, their use is limited in LMICs (McClure et al., 2014).

Due to the high incidence and fatal consequences of these abnormalities in low-resource settings, affordable perinatal monitoring solutions are required. One of the most widely used, yet affordable methods for perinatal screening is fetal heart rate (FHR) monitoring. This technique has contributed to reduce perinatal and maternal risks through identification of non-reassuring fetal status (Ayres-de Campos and Bernardes, 2010). Moreover, FHR has the potential for detecting IUGR (Nijhuis et al., 2000; Ferrario et al., 2009), as well as CHD complications (Cullen et al., 1992; Berghella et al., 2001).

FHR monitoring is commonly performed through Cardiotocography (CTG) based on one dimensional Doppler Ultrasound (DUS), that is also used in low cost (under $17) hand-held devices which can be operated by non-experts. This DUS-based low-cost device has been used to develop affordable perinatal monitoring systems, thus facilitating screening in LMICs. Stroux et al. introduced a mobile-health monitoring system, based on a low-cost transducer and operated by illiterate birth attendants, to detect fetal compromise, such as IUGR, in rural Guatemala (Stroux, 2016; Stroux et al., 2016). DUS can also provide more information beyond the FHR, such as the cardiac valve function, which can further facilitate detection of CHDs and assessment of the fetal development (Marzbanrad, 2015; Marzbanrad et al., 2016b).

Despite the benefits of 1D-DUS, it is susceptible to noise affecting its quality, and it is non-stationary due to the fetal movements, which can complicate the FHR monitoring. Since the quality of the recorded signals is critical to properly detect FHR abnormalities, the assessment of the signal quality is an essential part of the recording process. Stroux and Clifford reported that the accuracy of FHR analysis depends on the signal quality, hence the quality should be ensured during the data collection (Stroux and Clifford, 2013). Magenes et al. also showed the necessity of removing CTG signals with low quality before applying methods for detecting fetal anomalies (Magenes et al., 2001). The quality assessment process, enables providing feedback to the operator during data collection, allowing them to retake or exclude the low-quality signals.

To date, little work has been published concerning the quality assessment of the DUS signals. Stroux and Clifford proposed a method to validate the quality of DUS signals recorded using a hand-held device connected to a smart phone (Stroux and Clifford, 2014; Stroux, 2016). For this purpose, they extracted features based on sample entropy, wavelet decomposition coefficients, and the phone's triaxial accelerometer output. To assess the quality, a logistic regression and a support vector machine (SVM) were trained to classify the recordings into noisy and clean categories. The logistic regression model was able to classify the signal quality with an accuracy of 95.14% on test data, while the SVM achieved an accuracy of 94.44%. Marzbanrad et al. proposed an automated method to assess the DUS signal quality for the application of fetal valve motion identification (Marzbanrad, 2015; Marzbanrad et al., 2015). In their method, DUS signals were segmented into cardiac cycles using non-invasive fetal electrocardiogram (fECG) as reference. Then, 12 features including power, statistical and entropy-based measures, were extracted from a frequency range associated with the fetal cardiac valve motion. Using these features, the signals collected from 57 fetuses were classified as good and poor quality, using a na ive Bayes model. The accuracy of the classification was 86% using 10-fold cross validation.

In the current paper, to improve the quality assessment for perinatal monitoring, we propose a simpler template-based method using only the DUS signal recorded by a low cost device, thus facilitating its implementation in LMICs.

## 2. Methods

### 2.1. Database

**Figure 1:**
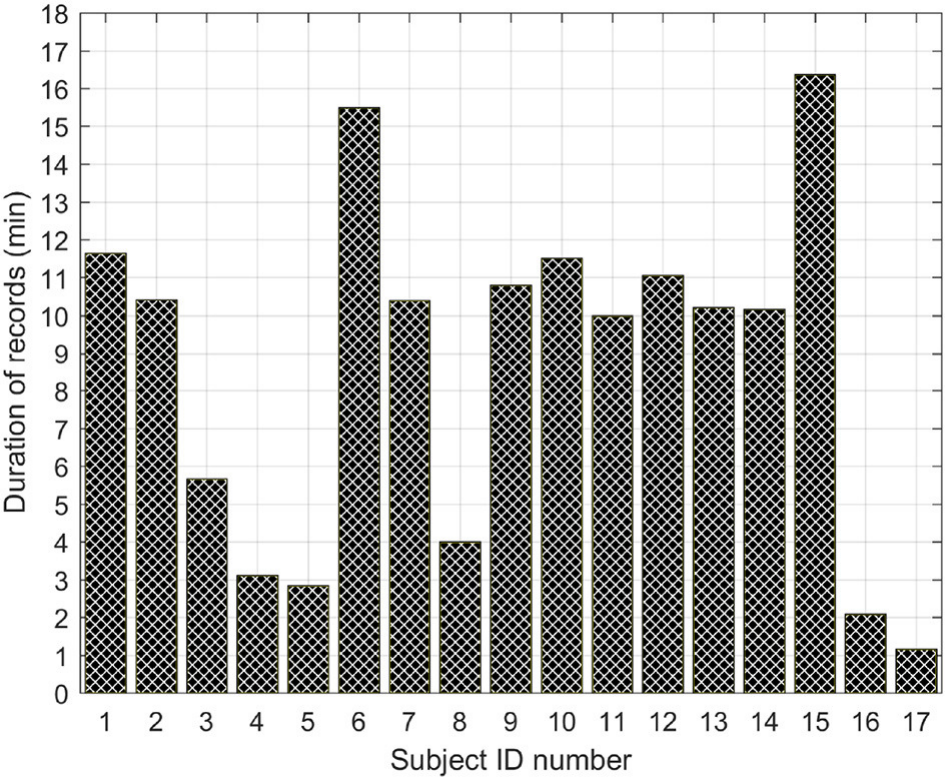
Duration in minutes of the total number of records per subject.

The DUS database used in this paper was collected at the John Radcliffe Hospital in Oxford as a part of the study presented in Stroux and Clifford (2014) and Stroux (2016). The study was approved by the NHS Health Research Authority, REC reference: 12/SC/0147 and written consent was obtained from each study subject prior to data collection. Each subject received detailed information on the study protocol and their right to withdraw from the study at any stage of the recording session. This database contained 1D-DUS signals recorded from 17 subjects at a sampling frequency of 44.1 KHz using a hand held transducer (AngelSounds Fetal Heart Detector, Jumper Medical Co., Ltd.) with an ultrasound frequency of 3.3 MHz. Subjects were women with singleton pregnancy, over the age of 18, who were scheduled for a routine CTG. The duration of recording per subject is shown in Figure 1.

### 2.2. Segment selection

Each of the 1 min-length DUS signals were labeled by three different annotators who had relevant experience in analyzing cardiac audio recordings for quality. Each reviewer independently labeled each second of the record as good or poor quality. After labeling, each record was split into segments of 3.75 with a 3 s sliding window (i.e., a 0.75 s overlap). The duration of 3.75 was fixed since it is the usual length for computerized analysis of fetal non-stress tests based on the Dawes/Redman criteria (Dawes et al., 1981; Pardey et al., 2002). To ensure that each segment belongs to only one class, only the segments with all their samples of the same class were kept. These segments were assigned to 4 different classes as follows:

- Good Quality: Three annotators labeled all the segment as good quality.
- Mostly clean: Two annotators labeled all the segment as good and one labeled all the segment as poor quality.
- Mostly noisy: One annotator labeled all the segment as good and two labeled all the segment as poor quality.
- Poor Quality: Three annotators labeled all the segment poor quality.

**Figure 2:**
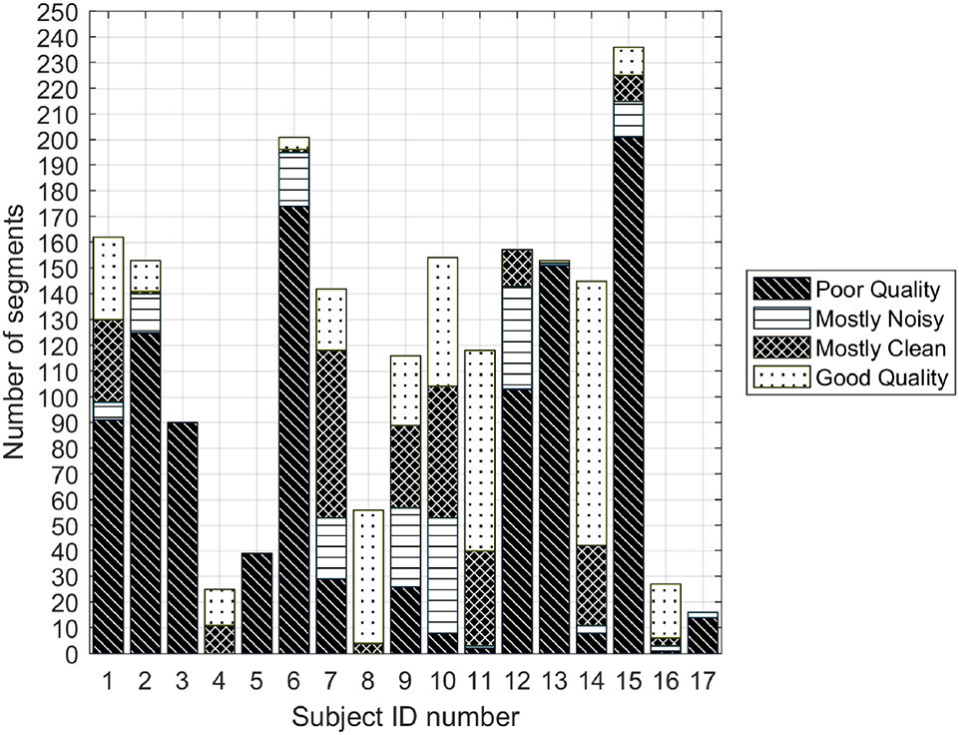
Number of poor and good quality 3.75 s segments for each of the subjects for which all three annotators agreed on labels.

A total of 1,990 segments (430 good, 1,062 poor, 292 mostly clean, and 206 mostly noisy quality) were identified. Figure 2 illustrates the balance of segments across patients. Note that the quality of the recorded signals varies from one patient to another and may change over a single recording session because we observed that for some recordings there are both good and bad quality segments.

The classifier in this work was only trained using poor and good quality segments. The rationale behind this stems from the fact that segments on which one or more experts cannot agree are not meaningful in reporting statistics, since we cannot categorize them in a single class. However, after training the classifiers, the optimal classifier was also evaluated with the mostly clean and mostly noisy segments to determine its capacity for detect intermediate quality segments.

### 2.3. Preprocessing

The DUS signals were resampled at 4,000 Hz using a least-square linear phase anti-aliasing filter. This downsampling does not affect the information content of the signal, since the fetal heart activity corresponds to the DUS signal frequencies below 1,650 Hz for a transducer of 3.3 MHz (Shakespeare et al., 2001). Hence, the Nyquist-Shannon sampling criterion was satisfied after downsampling.

### 2.4. Template-based quality assessment of 1-D doppler ultrasound

**Figure 3:**
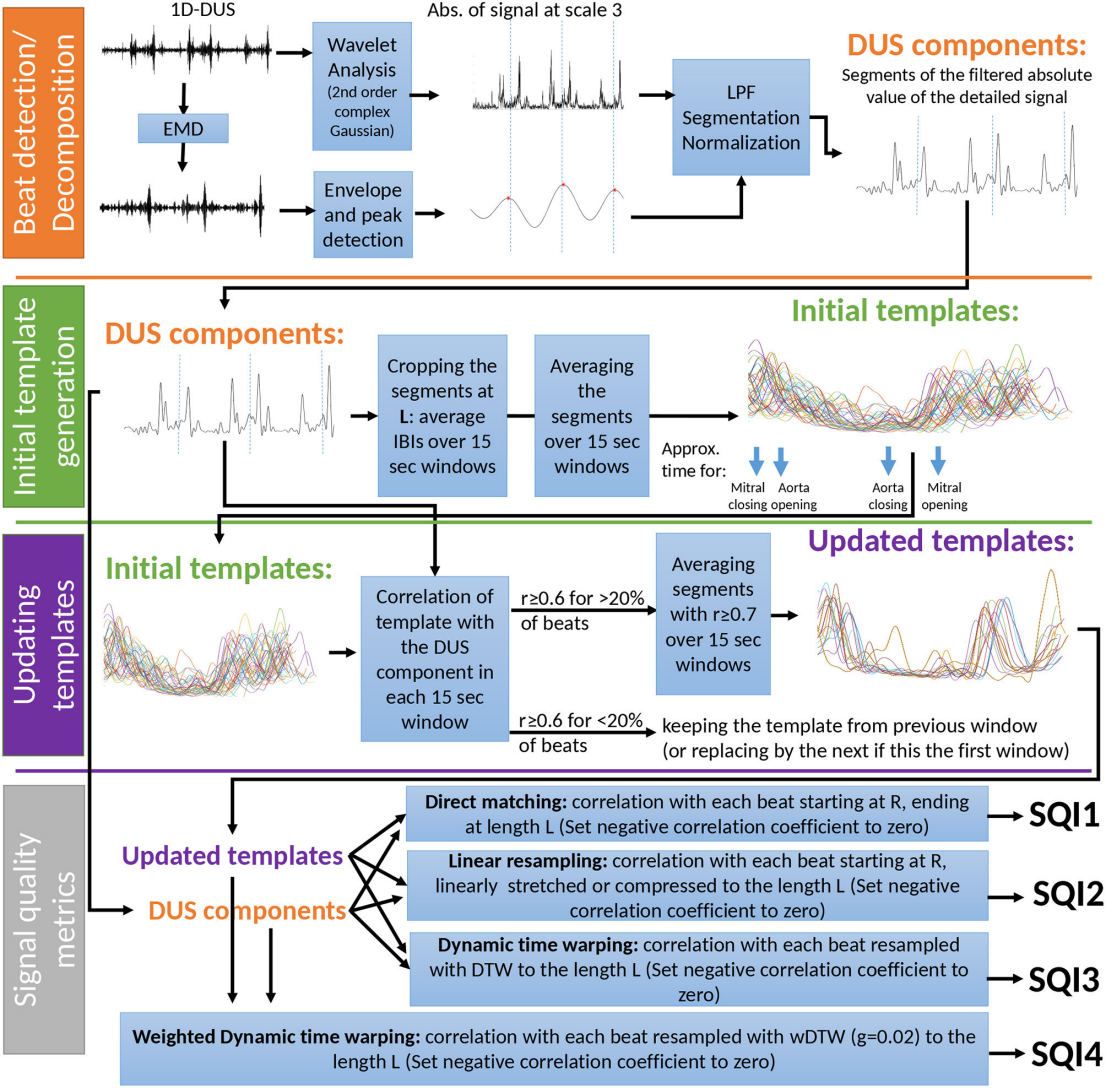
Overview of the template-based quality method for the Doppler signal.

To assess the quality of the DUS segment, a template-based algorithm was developed. This method consists of 4 stages (Figure 3). First, the beats of the DUS signals were estimated using Empirical Mode Decomposition (EMD). Then, using the estimated beats, templates for windows of 15 s were derived. These templates were then optimized in stage 3, and finally, the quality index of the DUS segment was calculated in stage 4. These stages are illustrated in Figure 3 and explained in the following sections.

This method, as all the remaining methods of this work, were implemented in Matlab and executed in a machine with a standard processor (Intel(R) Xeon(R) CPU E5-2660 v2 @ 2.20GHz).

#### 2.4.1. Beat detection

Individual cardiac cycles (or beats) were detected using EMD, based on a method presented in Marzbanrad et al. (2014). EMD is an empirical method for decomposing non-stationary and non-linear signals into a set of components called Intrinsic Mode Functions (IMFs). It is a data-driven method that is able to adapt to the signal properties without requiring a basis function, unlike other time-frequency decomposition methods (Huang, 2014). This characteristic allows EMD to properly analyze non-linear and non-stationary natural processes.

Each extracted IMF satisfies 2 properties: firstly, the number of maxima and minima and the number of zero crossing should differ at most by 1; secondly, the mean value between the envelope of the local maxima and the envelope of the local minima must be zero at any point. To obtain the IMFs, EMD uses an intuitive algorithm called “sifting procedure.” It is an iterative procedure, which finds all the IMFs of the signal until the difference between output and the input of the sifting procedure becomes a monotonic function. More details of the method can be found in Huang et al. (1998).

To find the beats from the DUS signals an algorithm was developed to allow switching between the first four IMFs, which were obtained over 4 s windows. For each of these IMFs, the peaks were detected based on the positive first derivative and negative second derivative criteria. Then, using the identified peaks, the IMFs were enveloped to obtain four IMF envelopes for each window. To detect the best envelope for segmentation, a metric based on the standard deviation of the peak to peak intervals (PPIs) was applied. Namely, the IMF with the minimum average of the standard deviation of PPIs was selected as the optimum IMF. To deal with the possible mismatching of the selected IMFs in adjacent windows, a short overlapping window of 1 s was used to correct missing or double identified peaks. The peaks of the optimum IMFs were selected as possible beat locations. These peak locations were further corrected through a moving windows of 5 PPIs, replacing the middle PPI by the average of the rest in the window, if they differed by more than 20% (Marzbanrad et al., 2016a). The corrected peaks were set as beat location, and were used to segment the 1D-DUS signal into Beat to Beat Intervals (BTBI).

Continuous Wavelet transform (CWT) was then applied to the DUS signal over 25 s windows. In this work, the CWT was applied using second order complex Gaussian function as Wavelet mother. Moreover, only the signal decomposed at scale 3 was selected since it was found that the 3rd level is the most relevant for detecting value movement. This scale contained frequencies below 1,000 Hz, which mainly reflect the fetal heart activity; valve motion is around 990 Hz for a transducer of 3.3 MHz (Murata and Martin Jr, 1977), and wall velocities between 257 and 429 Hz for a transducer of 3.3 MHz (Shakespeare et al., 2001).

After applying the CWT, the envelope of the absolute value of the decomposed signal was estimated by interpolating the maxima. This envelope was then smoothed using a low pass filter and segmented into cardiac cycles using the estimated beat locations. Each segment was normalized by subtracting its mean and dividing by its standard deviation. These normalized segment were used to generate the templates for the signal quality assessment.

#### 2.4.2. Initial template generation

Using the normalized cardiac cycle segments, the initial templates were calculated using a window of 15 s. The length (L) of the template was calculated as the average of BTBIs in each 15 s window. The initial template of the window was determined by averaging the segments starting at the beat location and lasting at length L. This procedure was repeated for each window, thereby obtaining an initial template for each window of 15 s.

#### 2.4.3. Updating templates

The initial template of each window was updated based on the correlation function. For each window, the correlation of the template and the segments starting in a beat location and lasting at length L was calculated. The window template was updated by averaging only the segments with a correlation (*r*) greater than or equal to 0.6. In case the initial template of a window did not have a correlation of *r* ≥ 0.6 for at least 20% of the beats, the template was assumed as invalid, and replaced by the one from the previous window. If the initial template of the previous window was invalid, the one from the next window was selected.

#### 2.4.4. Signal quality metrics

After updating templates for each window, the quality indices were calculated as the correlation of the segments with the template in their corresponding window. The correlations was calculated in four ways:

- **Direct matching SQI**. The segments of each 15 s window, beginning at the beat location and ending at the length of the template (L) were used to calculate the correlation coefficient with the template and this was denoted as SQI1. If the segment was shorter than L, it was padded by zeros.
- **Linear resampling SQI**. Each estimated beat of the window was linearly stretched or compressed, if the length of the beat was shorter or longer than L, respectively. Then, the correlation coefficient with the template was denoted as SQI2.
- **Dynamic time warping SQI**. Using Dynamic Time Warping (DTW), the segments were transformed to the length L [as performed in our earlier work (Li and Clifford, 2012)] and the correlation with templates was denoted as SQI3.
- **Weighted dynamic time warping SQI**. One drawback of DTW is that it gives too much freedom to the segment to adapt to the template. This was addressed by Jeong et al. who introduced the weighted DTW (wDTW) (Jeong et al., 2011). This method penalizes points with higher phase from the reference template by applying weights, thereby minimizing the distortion caused by outliers. In the current work, the parameter controlling the penalty was optimized through cross-validation to achieve the highest accuracy. The best value was found to be 0.02. The correlation of the transformed segments in the window with the corresponding template, was denoted SQI4.

For all methods, any negative values of these SQI (negative correlation) were set to zero.

### 2.5. Sample entropy and power spectrum density (PSD)

In addition to the Template-based SQIs, two other key features were estimated from the DUS signals. The first one was sample entropy (*H*_*s*_), which has shown a promising potential for discriminating between good and poor quality DUS segments (Stroux and Clifford, 2014). Sample entropy measures the regularity of a signal by finding reoccurring patterns in it. To this end, three parameters are defined: the length of the signal *N*, the pattern length *m* and the matching tolerance *r*. Sample entropy is defined as the negative logarithm of the probability that a time series of length *N* with reoccurring pattern of length *m* within a set tolerance of *r*, also has reoccurring patterns of length *m* + 1 under the same tolerance constraint. In this work, the sample entropy was calculated setting the parameters *m* = 2, and *r* as 0.1 times the standard deviation of the input time series. The entropy was calculated using the procedure described in Richman and Moorman (2000).

The second additional feature extracted was the Power Spectrum Density (PSD) ratio. This feature was used in order to the evaluate the power of the DUS signals at different frequency ranges. The range for calculating the ratio was determined using a grid search. Since cardiac movements are associated with a Doppler frequency range of 100 to 600 Hz using a 2 MHz transducer (Wheeler et al., 1987), which translate to a scaled range of 165 to 990 Hz for 3.3 MHz transducer, the ranges of values of the grid search were fixed from 80 to 400 Hz and from 580 to 900 Hz for the low and high frequency interval limits, respectively. For each possible pair of values, the capacity for discriminating between good and poor quality segments was measured using the earth mover's distance. The range with the highest earth mover's distance between the distribution of the ratios of good and poor classes was found to fall in the range 160–660 Hz. Thus, the PSD ratio of each DUS segment was calculated by dividing the power spectrum contained in the interval [160−660 *Hz*] by the total power, thereby measuring the percentage of power associated with cardiac movements.

### 2.6. Feature vectors

Applying the template-based method resulted in four different SQIs for each estimated beat of the segment, thus obtaining a total of 4*N*_*b*_ indices by segment, where *N*_*b*_ is the number of beats of the segment. As the number of beats varied for each segment, we selected the median value of each quality index of the segment as the final SQI. Thus, each segment had only one value for SQI1, SQI2, SQI3, and SQI4. Finally, the sample entropy and the PSD ratio were added to the feature vector, thereby obtaining a total of six features for each segment.

### 2.7. Classification

The above features were used to train an SVM classifier. SVM is a classifier that finds the best hyperplane that maximizes the margin between two classes. When the data are not linearly separable, a kernel function is used to transform the data to a different space in which the data can be separated. In this work, a Gaussian radial basis function kernel was used:

(1) $$k(x_{i},x_{j})=\exp\left(\frac{-|x_{i}-x_{j}{|}^{2}}{2\sigma^{2}}\right),$$

where *x*_*i*_ and *x*_*j*_ are feature vectors, and σ is a free parameter of the kernel. A large value of σ increases the bias but reduces the variance of the classifier and a small value causes the opposite effect. To find the best value for a given training set, σ is usually tunned using heuristic methods or a brute force search.

The class prediction, *y*, of a given feature vector, *x*, is calculated using the dual representation of SVM as:

(2) $$y=sgn\left(\sum_{i\;=\;1}^{N}\alpha_{i}y_{i}k(x_{i},x)+b\right),$$

where *x*_*i*_ is the *i-th* feature vector of the training set and *y*_*i*_ = [−1, 1] is its class; α ≥ 0 are Lagrange coefficients obtained by quadratic optimization; *b* is the intercept of the margin; and *k*(*x*_*i*_, *x*_*j*_) is the kernel function (Equation 1). The α coefficient is only greater than 0 for those points that are in the margin. These points are called support vectors. In addition to the parameters of Equation 2, SVM has a hyperparameter called the soft margin constraint (*C*). This parameter regularizes the margin allowing the cost function to ignore some points to establish an adequate margin for the training set. More details concerning the SVM can be found in Abe (2005). In this work, the SVM parameters *C* and σ were optimized using five-fold cross-validation and a grid search on the training set. The grid search was defined by *C* ∈ {2^−3^, 2^−1^, …, 2^5^} and σ ∈ {2^−5^, 2^−2^, …, 2^2^}.

### 2.8. Method performance assessment

To assess the performance of the method proposed here, the dataset was split into two equal subsets; the training and test sets. The the training set was used to determine the combination of features most relevant for assessing the quality of DUS segments. The test set provides an assessment of the accuracy on an independent dataset.

To split the dataset into two equal subsets (training and test sets), the subjects were ranked based on their number of good segments in descending order. Then, the data of each of the subjects were alternately assigned to the subsets. In other words, the first subject's samples were assigned to the training subset, the second ones to the test subset, the third one to the training subset, and so on. As the number of patients was odd (17), the samples of the last subject were assigned to the subset with the lowest number of segments.

The best combination of features was found by calculating the accuracy of all possible feature combinations on the training set. Since the dataset presented an imbalance among classes (Figure 2), the accuracy was calculated using stratified five-fold cross validation with bootstrapping. Specifically, the accuracy of each feature combination was determined as follows: subjects of the training set were split into 5 folds. For each fold, 120 signals (60 per class) samples from the subjects of the fold were randomly selected using sampling with replacement (bootstrapping). The selection was performed in proportion to the subjects' sample quantity in each fold. The rationale behind this validation process is that the bootstrap applied to the cross validation folds adjusts the class imbalances, which is a critical factor for SVM classifiers (Chawla et al., 2004). Moreover, as the cross validation did not assign samples of the same subject to different folds, it provided an unbiased accuracy estimation. To obtain a more reliable accuracy, the described validation process was repeated 100 times, assigning subjects into different folds at each repetition.

For each iteration of the five-fold cross validation, the training set was normalized by subtracting the mean of the respective feature vector and dividing by its standard deviation. The test set was normalized using both mean and standard deviation derived from the training data. The cross validation accuracy of each iteration was averaged by selecting the median of the 5 accuracy values of the folds. Likewise, the accuracy of the 100 repetitions was selected as the median of the 100 accuracy values. This procedure was performed for each combination of features.

In addition to the overall accuracy of the classifiers, the sensitivity and specificity were also estimated. Sensitivity was defined as the proportion of good quality segments properly classified, whereas specificity denoted the portion of poor quality segments correctly classified.

To determine the capacity of the method for predicting intermediate quality segments, a SVM classifier was trained with the good and poor segments of the test set using the most common parameters *C* and σ for the 100 bootstrap repetitions. Once the best combination of features was determined (maximizing accuracy, then specificity), the classifier was fixed and assessed on the test using the same bootstrap cross-validation validation procedure used for the training set. Finally, the probability of belonging to good class was also estimated for the mostly noisy, and mostly clean segments without retraining to assess the performance of the classifier on all data.

## 3. Results

### 3.1. Feature selection

Table 1 presents the median accuracy, sensitivity and specificity of the best combination of input features for up to 6 possible features. As can be seen, the classifier was able to classify the quality of a DUS segments with up to 85.8% accuracy using either the combination SQI2 and sample Entropy (*H*_*S*_), or the combination SQI2, PSD ratio and *H*_*S*_. The accuracy of these two combinations of features resulted in a statistically significant improvement over the use of only one feature, *H*_*S*_ (*p* < 0.05, one-sided Wilcoxon rank-sum test). The results for all possible combination of features are presented in the appendix (Table A1).

**Table 1:** Median classification performance of the 100 five-fold cross validation balanced with bootstrapping.

**Feature combination**	**Median accuracy ± IQR (%)**	**Median sensitivity (%)**	**Median specificity (%)**
*H*_*s*_	84.2 ± 5.8	100.0	78.3
SQI2,*H*_*s*_ ‡	85.8 ± 5.0	93.3	80.0
SQI2,PSD,*H*_*s*_	85.8 ± 5.0	83.3	90.0
SQI2,SQI4,PSD,*H*_*s*_	85.0 ± 8.3	85.8	88.3
SQI1,SQI2,SQI4,PSD,*H*_*s*_	84.7 ± 5.0	85.0	86.7
SQI1,SQI2,SQI3,SQI4,PSD,*H*_*s*_	83.8 ± 6.7	81.7	86.7

*IQR indicates inter-quartile range; ‡ indicates a significant improvement (Wilcoxon rank-sum test, P < 0.05) of a given feature combination compared to using a combination with one less feature*.

It can also be seen from Table 1 that the sensitivity tended to decrease with an increase in the number of features, whereas specificity steadily increase until three features were used. Since the combination of both two and three features leads to equivalent accuracy, the combination SQI2, PSD ratio and *H*_*S*_ was chosen to be evaluated on the test set, since this maximizes specificity, and reduces the chances that a poor quality segment is labeled as good quality.

### 3.2. Test set performance

Table 2 displays the accuracy, sensitivity and specificity of the combination of the SQI2, PSD ratio and *H*_*S*_ features. The median accuracy of this classifier using this combination was similar to the highest median accuracy achieved on the training set. However, the interquartile range for the test set was almost twice than that for the training set, indicating that the test set may exhibit a higher heterogeneity of features. Both sensitivity and specificity exceeded 90%.

**Table 2:** Performance of the classifier averaged over 100 five-fold cross validation runs balanced with bootstrapping for the test set (with) using SQI2, PSD ratio, and sample entropy (SQI2,PSD,*H*_*s*_) as features.

**Measure**	**Minimum (%)**	**1st Quantile (%)**	**Median (%)**	**3rd Quantile (%)**	**Maximum (%)**
Accuracy	65.8	79.2	85.8	90.0	96.7
Sensitivity	71.7	85.0	91.7	96.7	100.0
Specificity	61.7	89.3	91.7	95.0	98.3

### 3.3. Performance of classifier on intermediate quality segments

**Figure 4:**
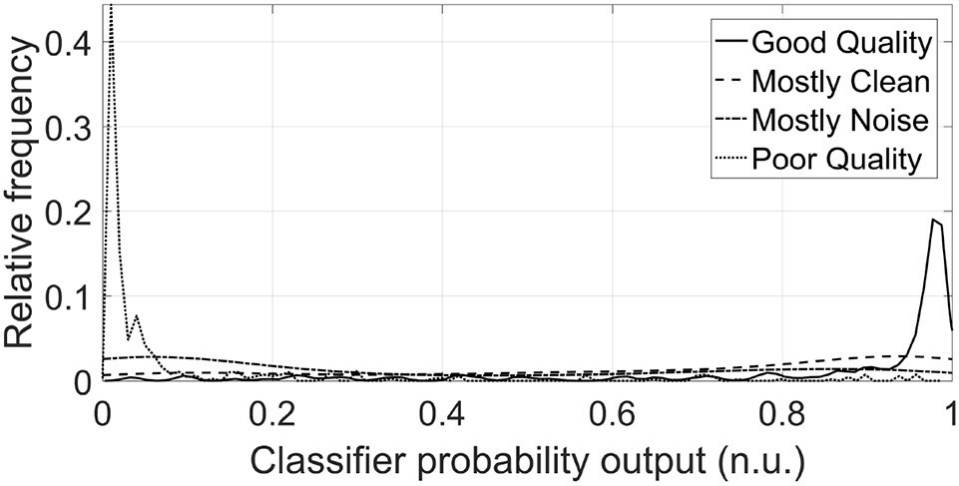
Distributions of classifier probability outputs for DUS segments of test set for each of the four classes (n.u. stands for normalized units). The threshold of belonging to the Good class was fixed at 0.56 for the classifier. The majority of the distribution of the Good and Mostly Clean classes lies above this threshold, whereas the majority of Poor and Mostly Noise classes lies below this threshold, as was expected. The probability distributions were smoothed using a normal kernel function (Bowman and Azzalini, 1997).

The classes of the mostly clean and mostly noise segments of the test set were also predicted using the same classifier [using SQI2, PSD ratio and sample entropy (*H*_*S*_) as features]. Note that these segments were not used in training. Figure 4 shows the relative distribution of output probabilities from the classifier of belonging to the good quality class for all four types of segments. The classifier established a probability threshold of 0.5575 for distinguishing between good quality and poor quality segments. The percentage of segments which lay above the threshold for good quality and mostly clean segments were 86.53 and 69.06%, respectively. On the other hand, the percentage of segments that lay below the threshold for poor quality and mostly noise segments were 96.50 and 63.69%, respectively.

## 4. Discussion

The results presented here suggest that it is possible to accurately classify the DUS quality using SQIs derived from DUS signals alone. Among the extracted features, sample entropy and PSD ratio provided suitable discrimination between good and poor quality segments, which is consistent with previous works (Stroux and Clifford, 2014; Marzbanrad et al., 2015). However, the addition of our proposed template-based method, particularly after linear resampling of the beats to match the running template (SQI2), provided a statistically significant improvement in accuracy (see Table 1). Either combinations SQI2 and *H*_*s*_ or SQI2, PSD ratio and *H*_*s*_ resulted in a statistically significant accuracy; nevertheless, in order to maximize specificity, the combination of SQI2, PSD ratio and *H*_*s*_ was selected for assessing the classifier on the test set.

The selected features achieved an accuracy of 85.8% on the test set, thus suggesting that this metric is suitable for quality assessment based only on DUS signals. Although this feature combination exhibited more variance on the test set than on the train set, the achieved accuracy indicates that the model was not overfitted, and its complexity of three features is viable for assessing DUS quality. Furthermore, the balance toward specificity provided by the three chosen features (SQI2, PSD ratio, and sample entropy) ensures a high number of good quality segments is preserved, as well as small number of false-positive segments.

The best combination of features also showed an adequate capacity for classifying segments associated with intermediate quality zones (mostly noisy and moistly clean segments). Although both mostly clean segments and mostly noisy segments exhibited a mostly flat distribution, their centers where more closer to the good quality center and poor quality centers, respectively as it was expected. Specifically, almost 70% of the mostly clean probabilities laid above the SVM prediction threshold, whereas higher than 63% of the mostly noise probabilities laid below the SVM prediction threshold. This discrimination ability for the two ambiguous classes indicates the potential of the approach outlined in this work.

Regarding the template-based SQIs, the EMD-based approach appeared to facilitate identification of beat intervals, since the correlation with each template was generally high. Small offsets in the relative start and end point of each beat were mitigated by the use of resampling prior to correlation. The segmentation facilitated beat-by-beat quality assessment, which is the first step toward detecting fetal abnormalities from DUS signals. The template optimization process obtained representative templates for quality assessment since the initial template was only averaged with those segments which exhibited a moderate or strong correlation (*r* ≥ 0.6) with the initial template (average of first N beats).

Although Stroux and Clifford reported a higher accuracy (95.14%) on the same database (Stroux and Clifford, 2014), their work cannot directly compared to the current work since different statistical validation approaches were used. Specifically, Stroux and Clifford trained on two thirds of the data set and held out one third for testing, with no cross validation. In this work, stratified five-fold cross validation was used with bootstrapping (repeated 100 times), with subject stratification across different folds in each repetition. The accuracy obtained in this work cannot directly compared to that of Marzbanrad et al. (2015) since they tested their method with a different dataset. However, our method can be compared to the aforementioned previous works by analyzing the effect of adding the index SQI2 to common features of the other works, namely, sample entropy. As was previously showed in Table 1, by using the SQI2 feature in addition to sample entropy, the accuracy statically significantly increased.

Another advantage of our method over previous works is that the proposed method does not need additional sources, such as accelerometer data (Stroux and Clifford, 2014) or an fECG signal (Marzbanrad et al., 2015) to assess the DUS quality. A key advantage of using only DUS signal is that the recording process is simple, facilitating the use of this technology by non-experts in low-resource settings. Finally, using only one source for quality assessment reduces health screening costs, facilitating its use in LMICs.

Despite the promising results, one limitation of the current method is that it was only tested using DUS signals recorded by professional midwives in hospital settings. The LMIC context is often severely resource constrained and there is a lack of widespread training for midwives, particularly in the use of technology. Consequently, signal quality is likely to be lower in recordings taken in LMICs. The noise content may also be different if the audio cable is not incorrectly inserted, introducing ambient sounds such as animals, extreme weather, and interference from non-hospital electronics. Nevertheless, the template-based method proposed here could be adjusted to specific conditions with a relevant training set.

Another possible limitation may be that the introduced method was only tested using one database labeled by three annotators. As DUS quality annotation is prone to inter-observer variability, testing the method with datasets annotated by different experts may reduce the accuracy. However, the high accuracy achieved by the combination of sample entropy, PSD ratio and SQI2 used in this work, provides optimism for the use of the template-based method for different datasets, especially with retraining.

## 5. Conclusion

The work presented in this article proposed a template-based method to assess the quality of 1D-DUS signals recorded by a low cost device. The introduced template-based indices provided a simpler method based on only DUS signals, thus facilitating its implementation in LMICs. The approach described in this work can provide the operator with an accurate and timely feedback on the quality of the recordings, to allow discarding the low quality signals in real time and prompt users to re-record data. Therefore, this quality assessment technique could potentially facilitate reliable fetal monitoring by non-experts toward reducing perinatal health burdens.

## Author contributions

CV implemented the second version of the method, performed experiments, analyzed results, wrote the paper. FM designed the method, implemented the first version of the method, she reviewed edited the paper. LS recorded the One Doppler Ultrasound signals used in this work. GC is the supervisor of CV; he guided the experiments performed by CV; he reviewed and edited the paper.

### Conflict of interest statement

The authors declare that the research was conducted in the absence of any commercial or financial relationships that could be construed as a potential conflict of interest.

## Appendix

**Table A1:** Median classification performance of the 100 five-fold cross validation balanced with bootstrapping for all the possible feature combinations.

**Feature combination**	**Median accuracy (%)**	**Interquartile range (%)**
*H*_*s*_	84.17	5.83
PSD	84.17	6.67
SQI4	64.17	11.25
SQI3	67.92	5.00
SQI2	74.17	6.67
SQI1	61.67	10.42
PSD,*H*_*s*_	85.06	5.00
SQI4,*H*_*s*_	84.58	5.00
SQI4,PSD	78.42	15.42
SQI3,*H*_*s*_	83.33	6.67
SQI3,PSD	84.17	12.92
SQI3,SQI4	65.00	9.58
SQI2,*H*_*s*_	85.83	5.00
SQI2,PSD	84.65	10.83
SQI2,SQI4	69.17	5.83
SQI2,SQI3	73.33	7.50
SQI1,*H*_*s*_	85.83	5.46
SQI1,PSD	81.67	8.33
SQI1,SQI4	61.67	9.17
SQI1,SQI3	62.50	7.22
SQI1,SQI2	71.67	8.75
SQI4,PSD,*H*_*s*_	81.67	8.75
SQI3,PSD,*H*_*s*_	85.00	7.50
SQI3,SQI4,*H*_*s*_	81.67	5.83
SQI3,SQI4,PSD	80.83	10.83
SQI2,PSD,*H*_*s*_	85.83	5.00
SQI2,SQI4,*H*_*s*_	84.17	5.00
SQI2,SQI4,PSD	83.33	8.33
SQI2,SQI3,*H*_*s*_	84.17	5.47
SQI2,SQI3,PSD	80.00	9.58
SQI2,SQI3,SQI4	67.50	8.33
SQI1,PSD,*H*_*s*_	85.00	6.67
SQI1,SQI4,*H*_*s*_	82.50	6.25
SQI1,SQI4,PSD	78.33	12.92
SQI1,SQI3,*H*_*s*_	83.33	5.00
SQI1,SQI3,PSD	82.50	14.58
SQI1,SQI3,SQI4	65.00	7.08
SQI1,SQI2,*H*_*s*_	85.00	5.83
SQI1,SQI2,PSD	84.17	8.33
SQI1,SQI2,SQI4	69.17	5.83
SQI1,SQI2,SQI3	72.50	6.67
SQI3,SQI4,PSD,*H*_*s*_	80.83	7.50
SQI2,SQI4,PSD,*H*_*s*_	85.00	8.33
SQI2,SQI3,PSD,*H*_*s*_	82.92	5.83
SQI2,SQI3,SQI4,*H*_*s*_	83.33	5.83
SQI2,SQI3,SQI4,PSD	77.08	9.17
SQI1,SQI4,PSD,*H*_*s*_	82.50	7.83
SQI1,SQI3,PSD,*H*_*s*_	85.00	10.83
SQI1,SQI3,SQI4,*H*_*s*_	79.17	5.00
SQI1,SQI3,SQI4,PSD	79.17	12.50
SQI1,SQI2,PSD,*H*_*s*_	84.17	6.25
SQI1,SQI2,SQI4,*H*_*s*_	82.50	5.83
SQI1,SQI2,SQI4,PSD	80.83	7.15
SQI1,SQI2,SQI3,*H*_*s*_	82.92	6.67
SQI1,SQI2,SQI3,PSD	79.17	8.75
SQI1,SQI2,SQI3,SQI4	68.33	7.08
SQI2,SQI3,SQI4,PSD,*H*_*s*_	80.83	10.00
SQI1,SQI3,SQI4,PSD,*H*_*s*_	77.50	10.83
SQI1,SQI2,SQI4,PSD,*H*_*s*_	84.65	5.00
SQI1,SQI2,SQI3,PSD,*H*_*s*_	84.17	6.67
SQI1,SQI2,SQI3,SQI4,*H*_*s*_	82.50	5.00
SQI1,SQI2,SQI3,SQI4,PSD	77.50	8.33
SQI1,SQI2,SQI3,SQI4,PSD,*H*_*s*_	83.75	6.67

*The table is grouped for feature vectors of the same length. For each combination of features, the median and interquartile range of the accuracy rate of the 100 repetitions are shown*.
